# Gaming experience between sexes might be the key differentiator in motor skill acquisition of a videogame task in adults

**DOI:** 10.14814/phy2.71166

**Published:** 2026-10-28

**Authors:** Zachary A. Riley, Quinn McCallion, Brach Poston

**Affiliations:** ^1^ School of Health and Human Sciences Indiana University—Indianapolis Indianapolis Indiana USA; ^2^ Department of Kinesiology and Nutrition Sciences University of Nevada‐Las Vegas Las Vegas Nevada USA

We read Dr. Rudroff's letter with great interest and always invite further discussion on the scientific paradigms and interpretations with motor learning. The central argument of Dr. Rudroff's letter is that the lack of statistically significant findings in our study (Blake et al., 2026) should not be interpreted as evidence that a‐tDCS does not influence motor learning in a guitar‐playing task. Instead, the results are more appropriately characterized as statistically indeterminate. Dr. Rudroff suggests that since our study did not show statistical differences with null hypothesis statistical testing and also did not show similarities with TOST equivalence analysis, that the “current analytical framework was insufficiently sensitive to detect structured heterogeneity in treatment response”.

Where does this leave the data? Is it in a statistical no‐man's‐land? To a certain extent, yes, the results were inconclusive due to the observed variability ruling out any meaningful or even trivial effects (Lakens et al., 2018). This leaves us with the task of either (1) moving on to a different research question or study paradigm as this high variability is likely to be present in any future studies, or (2) doing something to reduce the inherent variability in study task and provide a cleaner model for studying interventional effects. As stubborn researchers, we are always more likely to pursue the latter.

With regard to Dr. Rudroff's Dual Diversity Deficit concept (Rudroff et al., 2020), he is correct in the assertion that we sought population‐level demographic homogeneity by recruiting subjects for the study that were young (age 22.0 ± 3.1 years), healthy, and from a single university setting. We also recruited both males and females, which could be viewed as introducing neurobiological heterogeneity. However, our concern was less with the neurobiological heterogeneity (males vs. females) and more with the likelihood that we had a homogenous group of individuals with some level of gaming experience, thereby reducing the variability we expected to observe in learning the task. This is purposeful when studying an intervention such as a‐tDCS, to determine what impact the a‐tDCS had on learning and not what effect previous gaming experience had on learning.

Dr. Rudroff did a good job of summarizing some of the sex differences that exist between males and females and indicated that these are not minor sources of variance. However, his letter did not consider the previous experience the subjects had with the task or similar tasks. We also did not realize this until after the dataset was collected, as the amount of gaming experience was disparate between sexes. When collapsed across the a‐tDCS and SHAM groups, males averaged 6.54 ± 4.81 h/week of gaming, while females only averaged 1 ± 1.57 h/week. We stated in the original published paper that the weekly gaming experience of the 40 subjects (both sexes) broke down into: 0 h (*n* = 12), 0–1 h (*n* = 8), 1–3 h (*n* = 5), 3–6 h (*n* = 7), 6–10 h (*n* = 5), and >10 h per week (*n* = 3). Of these female subjects, 9 had no weekly gaming experience (of the 12 total) and none of the females had greater than 3–6 h weekly. This resulted in females averaging about ~10% fewer notes hit in their initial trial than males, and that difference remaining in their final trial, despite improving overall. The issue with the current study design is that we did not have enough total subjects to further parse into sex differences.

This brings us back to the original point of doing something to reduce the inherent variability in the study task and provide a cleaner model for investigation. One way of doing this would be to replicate the study with many more subjects to enable sex comparisons when the sexes have different amounts of gaming experience, and to use gaming experience as a covariate since we now know it could alter the interpretation of the treatment (a‐tDCS) effect. This is theoretically different from our study, and adding it in post‐hoc would inflate type I error rates. To Dr. Rudroff's point, the better option in the current situation would be to recruit many more male and female subjects but use stratified randomization of gaming experience to ensure we would be comparing similarly practiced individuals. Then the groups could have been balanced for gaming experience across not only conditions (tDCS vs. Sham) but also sexes.

As with many exploratory investigations, several factors that may have contributed to variability became apparent only after examination of the study findings. We thank Dr. Rudroff for further pointing out the reality of research in both the statistical and neurobiological domains: the study didn't fail; it just taught us something we didn't know when we started it.

## AUTHOR CONTRIBUTIONS

Z.A.R., Q.M., and B.P. contributed equally to the writing and revision of this letter to the editor, and all authors had a major contribution to the original published paper.

## ETHICS STATEMENT

None.
